# The role of chondrocyte-to-osteoblast trans-differentiation in fetal bone dysplasia of mice caused by prenatal exposure to dexamethasone

**DOI:** 10.3389/fphar.2023.1120041

**Published:** 2023-03-02

**Authors:** Jiayong Zhu, Xiaoqi Zhao, Hui Wang, Hao Xiao, Liaobin Chen

**Affiliations:** ^1^ Division of Joint Surgery and Sports Medicine, Department of Orthopedic Surgery, Zhongnan Hospital of Wuhan University, Wuhan, China; ^2^ Department of Pharmacology, Wuhan University School of Basic Medical Sciences, Wuhan, China; ^3^ Hubei Provincial Key Laboratory of Developmental Originated Disease, Wuhan, China; ^4^ Joint Disease Research Center of Wuhan University, Wuhan, China

**Keywords:** dexamethasone, trans-differentiation, hypertrophic chondrocytes, osteoblasts, bone dysplasia

## Abstract

Maternal exposure to dexamethasone can cause developmental toxicity of long bones in offspring. However, the effect of dexamethasone on the trans-differentiation of growth plate chondrocytes into osteoblasts and its role in bone dysplasia of fetuses caused by prenatal dexamethasone exposure (PDE) remains unclear. In this study, pregnant mice were treated with different doses, stages, and courses of dexamethasone according to clinical practice to reveal the phenomenon. Further, growth plate chondrocytes were treated with dexamethasone *in vitro* to clarify the phenomenon and mechanism. The results showed that PDE caused dysplasia of fetal long bones in female and male mice, accompanied by the delayed formation of the primary ossification center and the widening hypertrophic zone of growth plate cartilage. Meanwhile, PDE increased the number of hypertrophic chondrocytes at growth plate cartilage and decreased the number of osteoblasts at the primary ossification center. Moreover, PDE significantly decreased the expression of osteogenic transcription factor Runx2 but increased the expression of hypertrophic chondrocytes marker Col10. These above phenomena were more significant in the high dose, early stage, and double courses of dexamethasone exposure groups, and the male fetal mice showed more obvious than the female fetal mice. *In vitro*, dexamethasone significantly inhibited the trans-differentiation of growth plate chondrocytes into osteoblasts, accompanied by a decrease in Runx2 expression and an increase in Col10 expression. In conclusion, this study revealed the phenomenon and mechanism of fetal bone dysplasia caused by PDE from the new perspective of trans-differentiation disorder of growth plate chondrocytes to osteoblasts.

## Highlights

1. Dexamethasone caused fetal bone dysplasia with doses, stages, courses, and sex differences;
2. Dexamethasone inhibited trans-differentiation of growth plate chondrocytes to osteoblasts;
3. Trans-differentiation disorder of chondrocytes to osteoblasts contributed to fetal bone dysplasia.

## 1 Introduction

Dexamethasone, a synthetic glucocorticoid, can enter fetal blood circulation through the placenta to promote fetal lung maturation and reduce the incidence of respiratory distress syndrome. Therefore, dexamethasone is widely used in the clinical treatment of diseases related to threatened preterm birth. In clinical practice, dexamethasone is generally given to pregnant women with a preterm birth tendency at 24 weeks but less than 34 weeks of gestation (Jobe and Goldenberg, 2018). Globally, 15 million preterm infants are born every year, accounting for about 1/10 of live neonates (Lawn et al., 2013). Most of these preterm infants receive dexamethasone treatment before delivery to prevent the occurrence of respiratory distress syndrome (Vogel et al., 2014; Vogel et al., 2016; Chawanpaiboon et al., 2019). However, the use of dexamethasone during pregnancy has a “double-edged sword” effect. Previous studies have found that although treatment with dexamethasone during pregnancy can effectively reduce perinatal mortality, dexamethasone is the exact cause of developmental toxicity in the offspring (Xu et al., 2011; Murphy et al., 2012). Clinical studies have found that even the dose of dexamethasone recommended by the US Department of Health during pregnancy can lead to an increase in the incidence of low birth weight in newborns (Bloom et al., 2001; Sahoo and Aradhya, 2020). The developmental toxicity of bone caused by dexamethasone has been a wide concern for researchers. Clinical studies have shown that prenatal treatment with dexamethasone is an adverse factor in the reduction of bone mass and bone density in postnatal offspring (Kurl et al., 2000; Eelloo et al., 2008). We previously demonstrated that prenatal dexamethasone exposure (PDE) caused development retardation of long bones in fetal rats, manifested by the shortened bone length and inhibited formation of ossification center, and this development retardation even extended to postnatal life, leading to low peak bone mass and susceptibility to osteoporosis (Xiao et al., 2018; Wen et al., 2020; Shangguan et al., 2021). Recent studies showed that the trans-differentiation of chondrocytes to osteoblasts in growth plates played a vital role in the process of bone development (Yang et al., 2014; Tiffany and Harley, 2022). However, does dexamethasone adversely affect the trans-differentiation of growth plate chondrocytes into osteoblasts? Does this adverse impact participate in bone dysplasia caused by PDE? These scientific problems have not been reported.

The bone development of extremities is mainly accomplished through endochondral ossification and this process continues throughout the fetal to the postnatal period. In the process of endochondral ossification, continuous ossification of growth plate cartilage makes the long bone extend to both ends and form bone mass (Long and Ornitz, 2013). Growth plate chondrocytes undergo terminal differentiation and become hypertrophic during ossification, and some of these hypertrophic growth plate chondrocytes are eliminated by apoptosis or autophagy (Hallett et al., 2019). However, recent studies have shown that in the process of endochondral ossification, most chondrocytes in the growth plate become mature and do not enter apoptosis after hypertrophy, but directly transdifferentiate into osteoblasts (Li et al., 2018). For example, it was found that growth plate chondrocytes are the direct source of osteoblasts during bone development in the mouse model, and the trans-differentiation of chondrocytes into osteoblasts is necessary for trabecular bone formation in the embryonic and neonatal stages (Pazzaglia et al., 2020). In addition, osteoblasts transdifferentiated by chondrocytes from hypertrophic growth plates accounted for about 60% of all osteoblasts during endochondral ossification (Zhou et al., 2014). These results suggested that the trans-differentiation of growth plate chondrocytes into osteoblasts may play an important role in prenatal dexamethasone-induced bone dysplasia in fetal offspring.

Therefore, this study intended to systematically reveal the role of chondrocyte-to-osteoblast trans-differentiation in fetal bone dysplasia caused by PDE through establishing fetal mice models exposed to various doses, stages, and courses of dexamethasone. *In vitro*, primary growth plate chondrocytes were treated with different concentrations and times of dexamethasone to clarify the phenomenon and mechanism. This study elucidated the mechanism of the bone developmental toxicity of dexamethasone from a new perspective of chondrocyte-to-osteoblast trans-differentiation and provided a new idea for exploring the early prevention and treatment of dexamethasone-induced bone developmental toxicity.

## 2 Materials and methods

### 2.1 Drugs and reagents

Dexamethasone sodium phosphate injection (No. H42020019) was purchased from Shuanghe Pharmaceutical Co., Ltd. (Wuhan, China). Isoflurane was purchased from Baxter Healthcare Co. (Deerfield, IL, United States). TRIzol kit was purchased from Omega Bio-Tek (Doraville, GA, United States). Reverse transcription and quantitative PCR (RT-qPCR) kits were purchased from Takara Biotechnology Co., Ltd. (Dalian, China). Oligonucleotide primers for the gene used for analysis were synthesized by Sangon Biotech Co., Ltd (Shanghai, China). Fetal bovine serums were supplied by Gibco (St. Louis, MO, USA). Information on polyclonal antibodies is as follows: Glyceraldehyde 3-phosphate dehydrogenase (GAPDH) (No. AC033), The antibodies for Runx2 (A2851) were purchased from Abclonal Biotech Co., Ltd. (Wuhan, China). Collagen 10 (Col10) (abs153415) was purchased from Absin Bioscience Inc. (Shanghai, China). Secondary Antibodies information is as follows: Cy3 Goat Anti-Rabbit IgG (H + L) (AS007), FITC Goat Anti-Rabbit IgG (H + L) (AS011). Other chemicals and agents were of analytical grade.

### 2.2 Animal handling

Healthy male and female Kunming mice with SPF grade were purchased from Spefu (Beijing) Biotechnology Co., LTD. (License No: SCXK 2019-0010). Animal experiments were conducted at the Animal Laboratory Center of Wuhan University (Wuhan, China), which is accredited by AAALAC International (Association for Assessment and Certification of Laboratory Animal Management). All animal experimental procedures were performed following the Guidelines for the Care and Use of Laboratory Animals of the China Animal Welfare Committee. The protocol was approved by the Animal Experimentation Ethics Committee of Wuhan University School of Medicine. After 7 days of adaptive feeding, these mice were caged at 18:00 according to the ratio of male to female = 2:1, and the vaginal secretions of the female mice were smeared at 7:00 a.m. the next morning. The sperm observed under the light microscope was recorded as gestational day 0 (GD0).

**FIGURE 1:**
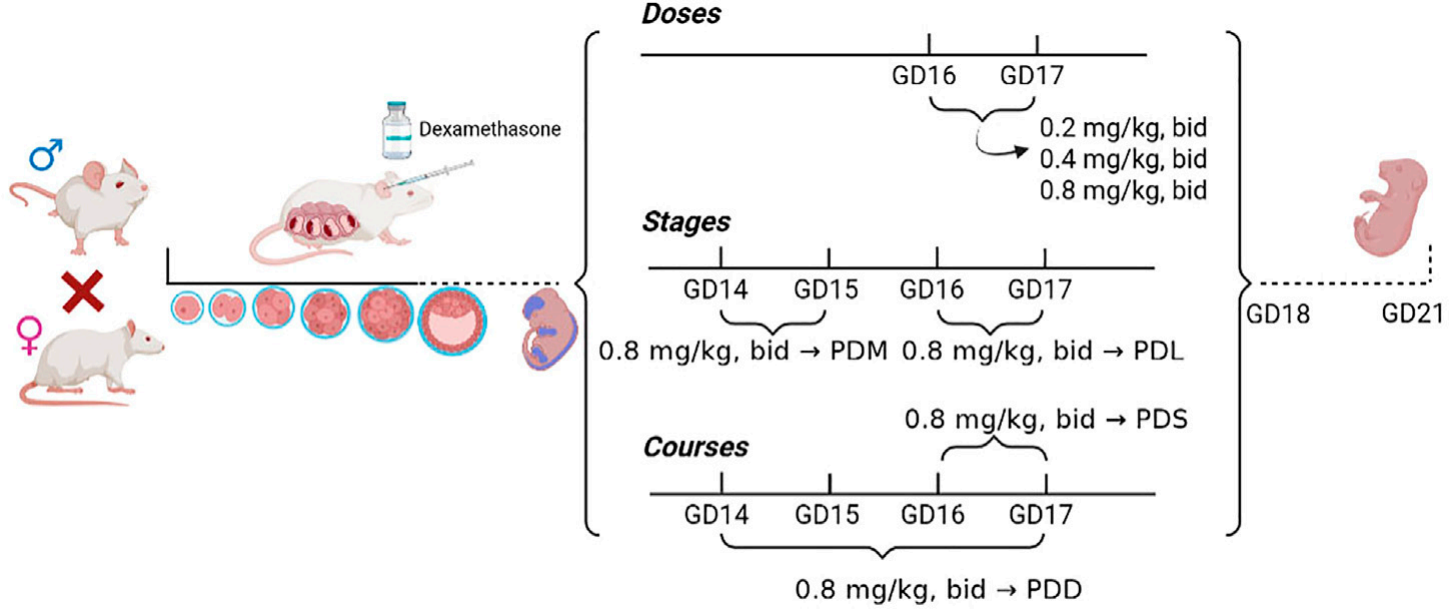
Flow chart of animal experiment processing. PDS, single course of dexamethasone exposure during pregnancy; PDD, double course of dexamethasone exposure during pregnancy; PDM, dexamethasone exposure in the middle-late trimester; PDL, dexamethasone exposure in the late trimester; GD, gestational day.

Pregnant mice were randomly divided into the control group and the dexamethasone treatment group (n = 12 per group) with different doses, stages, and courses (Figure 1). ① Groups treated with different doses of dexamethasone: the low-dose group (0.2 mg/kg, twice per day, 12 h interval, GD16-17 administration), the medium-dose group (0.4 mg/kg, twice per day, 12 h interval, GD16-17 administration), the high-dose group (0.8 mg/kg, twice per day, 12 h interval, GD16-17 administration); ② Dexamethasone treatment group at different stages: the early stage group (0.8 mg/kg, twice per day, 12 h interval, GD14-15 administration), the late stage group (0.8 mg/kg, twice per day, 12 h interval, GD16-17 administration); ③ Groups treated with different courses of dexamethasone: the single course group (0.8 mg/kg, twice per day, 12 h interval, GD14-15 administration), the double courses group (0.8 mg/kg, twice per day, 12 h interval, GD14-15 and GD16-17 administration). All the control group was given the same volume of normal saline subcutaneously under the same treatment. Pregnant mice in the above control group and dexamethasone treatment group were anesthetized with 2%–3% isoflurane on GD18 and sacrificed. Eight pregnant mice with litter sizes ranging from 8 to 14 were selected for the experiment. The bilateral lower limbs of the offspring mice were removed under a dissecting microscope, and the right limb was fixed in 4% paraformaldehyde solution for morphological examination, while the left limb was stored in a −80°C refrigerator for gene detection.

### 2.3 Isolation of growth plate chondrocytes and osteogenic differentiation

Primary growth plate chondrocytes were isolated as described previously (Maes et al., 2004; Wang et al., 2021). In brief, chondrocytes were isolated from growth plates of the proximal tibia and distal femur of 5-day-old mice. After removal of the perichondrium, isolated growth plates were pre-digested on a shaker for 30 min at room temperature with 0.1% collagenase type II (Gibco) dissolved in culture medium (DMEM/F12 medium supplemented with 10% fetal calf serum (FCS), 100 units/ml penicillin, 50 μg/ml streptomycin, 50 μg/ml ascorbic acid, and 100 μg/ml sodium pyruvate; all from Gibco). The remaining growth plate fragments were subsequently digested in 0.2% collagenase type II for 3 h on an incubator shaker at 37°C. The cell suspension obtained from the second digest was filtered through a 40 µm nylon mesh and single cells were recovered by centrifugation. Primary chondrocytes were seeded at a density of 5 × 10^5^ cells/cm^2^ and the medium was changed every other day. For induction of osteogenic differentiation, third-passage growth plate chondrocytes were seeded in 6-well plates and treated with osteogenic induction medium (α-MEM with 10% FCS, 100 μg/ml streptomycin, 100 U/mL penicillin, 10 mM β-glycerophosphate, 50 μg/ml ascorbic acid, and 10 nM dexamethasone). Then, the cells were treated with various concentrations of dexamethasone for further analysis.

### 2.4 Histological analysis

For histological analysis, sections of fetal femurs were stained with Safranin O. Olympus AH-2 optical microscope (Olympus, Tokyo, Japan) was used to obtain the photographs. A light imaging system (H550S, Nikon, Tokyo, Japan) was used to measure the length of the femur, the length of the hypertrophy zone (HZ) in growth plate cartilage, and the number of hypertrophic chondrocytes. For Von Kossa staining, the sections were dewaxed and stained with 5% AgNO_3_ until they became dark brown. A light imaging system (H550S, Nikon, Tokyo, Japan) was used to measure the length of the femur, and the length of the primary ossification center (POC). For alkaline phosphatase (ALP) staining, sections were cut (5 μm thick) and stained using an ALP staining kit (Jiancheng Bioengineering Institute, Nanjing, China). ALP + cells at the trabecular bone surface counted at ×100 magnification.

### 2.5 Immunofluorescence staining

The lower limbs were fixed in a 4% paraformaldehyde solution for 3 days and treated with a paraffin section. The tissue was cut into 5-μm thick sections for morphological staining analysis. In simple terms, paraffin sections were dewaxed to water and an antigen repair buffer containing EDTA (pH 8.0) was used for antigen repair. BSA was closed and a primary antibody was added. The dilution ratio of the primary antibody was anti-Col10 (1:200) and anti-Runx2 (1: 200), then the slices were placed flat in a wet box at 4°C and incubated overnight. On the next day, the second antibody of the corresponding species of the first antibody was added to cover the tissues, and the tissues were incubated at room temperature for 50 min away from light. The nuclei were re-stained with fluorescent Cy3 Goat Anti-Rabbit IgG (H + L) (AS007), FITC Goat Anti-Rabbit IgG (H + L) (AS011), and non-fluorescent DAPI and sealed with anti-fluorescence quenching tablet. All of the images were captured and then analyzed using the Nikon NIS Elements BR light microscope (Nikon, Tokyo, Japan). The staining intensity was determined by measuring the integrated optical density (IOD) in 10 different visual fields of each sample.

### 2.6 ALP staining and alizarin red staining

After 14 days of culture, the cultured cells were fixed in 95% ethanol for 30 min. ALP staining was performed using an alkaline phosphatase chromogenic kit according to the manufacturer’s protocol. They were then washed with PBS and observed by an inverted microscope (Olympus, Japan). Positive staining is represented as a purple area. The positive staining was quantitated by optical density measurement at 405 nm. For alizarin red staining, the cultured cells were fixed in 95% ethanol for 30 min and then stained with 0.1% alizarin red staining solution (pH 7.4) for 30 min at room temperature. And then, we employed an inverted microscope (Olympus, Japan) for observing and photographing the staining results. Positive staining is represented as red color mineralization nodules. The mineralization nodules were quantitated by optical density measurement at 562 nm.

### 2.7 Immunofluorescence staining of cells

Growth plate chondrocytes cultured in Confocal Dish were rinsed with PBS and fixed with 4% paraformaldehyde fixative for 15 min. Then they were permeated with 0.5% Triton X-100 at room temperature for 15 min. After washing with PBS, normal goat serum was dropped and sealed at room temperature for 30 min. Discard the sealing liquid, add the primary antibody directly according to the dilution ratio suggested in the primary antibody manual, and place it in a wet box at 4°C overnight. The next day, TBST was used for a full washing and fluorescence secondary antibody was added, and the cells were incubated at room temperature for 1 h without light. After that, PBST was used for washing, and DAPI was dropped and incubated for 5 min to avoid light. PBST was used for washing again, and images were observed and collected under a confocal microscope (Smart Proof 5, Carl Zeiss, Germany). The fluorescence intensity was determined by measuring the integrated optical density (IOD) in 10 different fields of each sample.

### 2.8 Overexpression of Runx2 in growth plate chondrocytes

Runx2 was ligated into pcDNA3.1 at the NheI and KpnI sites to construct pcDNA3.1. Runx2 vector. Prior to transfection, growth plate chondrocytes were seeded in six-well plates. 24 h later, the cells were then transfected respectively with pcDNA3.1 Runx2 vector, empty pcDNA3.1 vector or empty control vector using Lipofectamine 3,000 according to the manufacturer’s protocol. Eight hours later, the medium was exchanged for a fresh medium, and the cells were treated with 1,000 nM dexamethasone. The cells were harvested after 3 days for further analysis.

### 2.9 Total RNA extraction and RT-qPCR

The total RNA of cartilage tissues and primary chondrocytes were isolated using a TRIZOL reagent following the manufacturer’s protocol. The isolated RNA was stored at −80°C in aliquots. A total of 1 μg of purified RNA was reverse transcribed using a first-strand cDNA synthesis kit. The cDNA was amplified using a one-step RT-qPCR reaction. The RNA was assayed for Col10, Runx2, and GAPDH. The primer sequences for the mice are shown in Table 1. All of the cDNA sequences were obtained from the NCBI Entrez nucleotide databases, and the primers were designed using Primer Premier 6.0 (PREMIER Biosoft International, CA). Each of the designed primer sequences was queried using the NCBI BLAST database for homology comparison to determine the final primers used in the study.

**TABLE 1:** Primer used for a real-time quantitative polymerase chain reaction.

Genes	Forward primers	Reverse primers	Annealing
GAPDH	GGG​GTC​CCA​GCT​TAG​GTT​CA	CCC​AAT​ACG​GCC​AAA​TCC​GT	60°C, 30s
Col10	GCA​TCT​CCC​AGC​ACC​AGA​ATC	GCT​AGC​AAG​TGG​GCC​CTT​TA	60°C, 30s
Runx2	TCTGCTTGGCGGTGGC	TCC​GAG​GGC​TAC​AAC​CTT​GA	62°C, 30s

GAPDH, glyceraldehyde 3-phosphatedehydrogenase; Runx2, Runt-related transcription factor 2; Col10, Collagen Type 10.

### 2.10 Statistical methods

Prism Graphics (GraphPad Software, La Jolla, CA, United States, version 9.0) was used for all data analysis. Quantitative data expressed as the mean ± S.E.M. The data from *in vitro* experiments with different drug concentrations were analyzed using a one-way analysis of variance (ANOVA) with a *post hoc* test for multiple comparisons. In vivo experiment, we used unpaired, two-tailed Student’s t-tests for comparisons between control and treatment groups. Statistical significance was considered at *p <* 0.05 for all the tests.

## 3 Results

### 3.1 Effects of different doses of dexamethasone exposure on bone development and chondrocyte-to-osteoblast trans-differentiation in fetal mice

**FIGURE 2:**
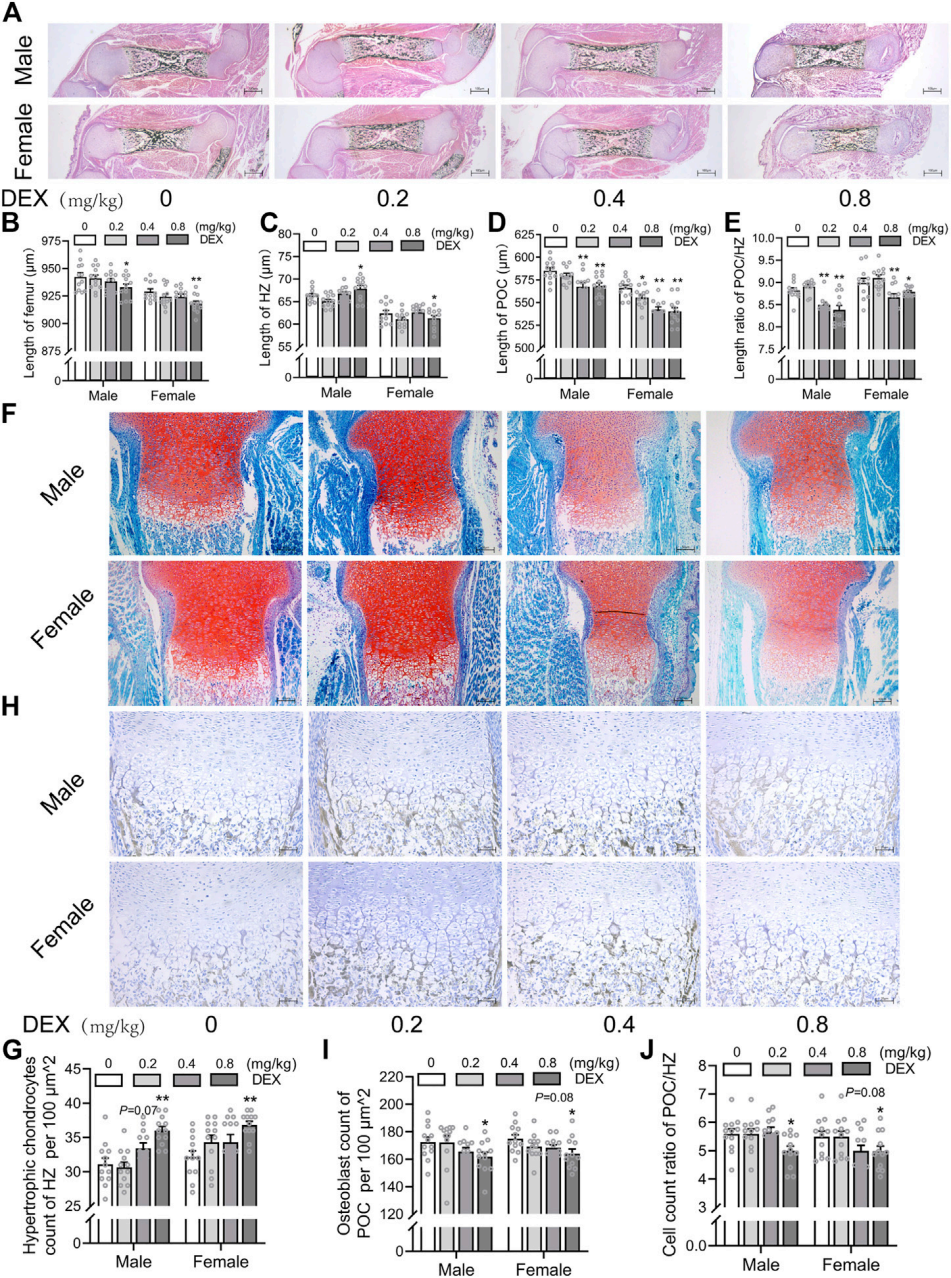
Effects of different doses of dexamethasone exposure on bone development and chondrocyte-to-osteoblast trans-differentiation in fetal mice. **(A)**. Typical images of vonkossa staining in fetal bone tissue; **(B)**. Quantitative analysis of fetal femur length; **(C)**. Quantitative analysis of the width of hypertrophy zone (HZ) in growth plate; **(D)**. Quantitative analysis of the length of the primary ossification center (POC); **(E)**. The ratio between the length of the POC and the width of HZ in the growth plate cartilage; **(F)**. Typical images of fetal bone tissue stained with saffron O and solid green; **(G)**. Analysis of hypertrophic chondrocytes number in growth plate cartilage; **(H)**. Typical images of alkaline phosphatase (ALP) staining in fetal bone tissue; **(I)**. Analysis of osteoblasts number in the POC; **(J)**. The relative ratio of the number of osteoblasts in POC to the number of hypertrophic cells in growth plate cartilage. ^*^ *p* < 0.05*,* ^**^ *p* < 0.01*,* 0 *v.s.*0.2 or 0.4 or 0.8 mg/kg.

To investigate the effects of different doses of PDE on bone development and trans-differentiation of growth plate chondrocytes into osteoblasts in fetal mice, we treated pregnant mice with different doses of dexamethasone (0.2, 0.4, 0.8 mg/kg) according to the therapeutic dose of dexamethasone in clinical. On gestational day 18, the pregnant mice were sacrificed and the fetal bone tissues were obtained for morphological staining. We observed that PDE shortened femoral length in fetal mice, especially in the high-dose dexamethasone exposure group, and this reduction in males was more significant than in females (*p <* 0.05, *p <* 0.01, Figures 2A,B). Then, we quantitatively analyzed the length of HZ and POC in the fetal bone tissue. The results showed that PDE increased the length of HZ in growth plate cartilage and inhibited the formation of POC in a dose-dependent manner (*p <* 0.05, *p <* 0.01, Figures 2C,D). Meanwhile, PDE reduced the ratio of POC length to HZ length (*p <* 0.05, *p <* 0.01, Figure 2E). Furthermore, we quantitatively analyzed the number of hypertrophic chondrocytes and osteoblasts in fetal mice. It was found that PDE dose-dependently increased the number of hypertrophic chondrocytes in the growth plate cartilage (*p <* 0.01, Figures 2F,G). Nevertheless, the number of osteoblasts in the POC was reduced by PDE in a dose-dependent manner (*p <* 0.05, Figures 2H,I). The cell number ratio of osteoblasts to hypertrophic chondrocytes was reduced by PDE (*p <* 0.05, Figure 2J). Taken together, PDE dose-dependently caused dysplasia of long bones in fetal mice, which is related to the trans-differentiation inhibition of hypertrophic chondrocytes into osteoblasts.

### 3.2 Effects of different stages of dexamethasone exposure on bone development and chondrocyte-to-osteoblast trans-differentiation in fetal mice

**FIGURE 3:**
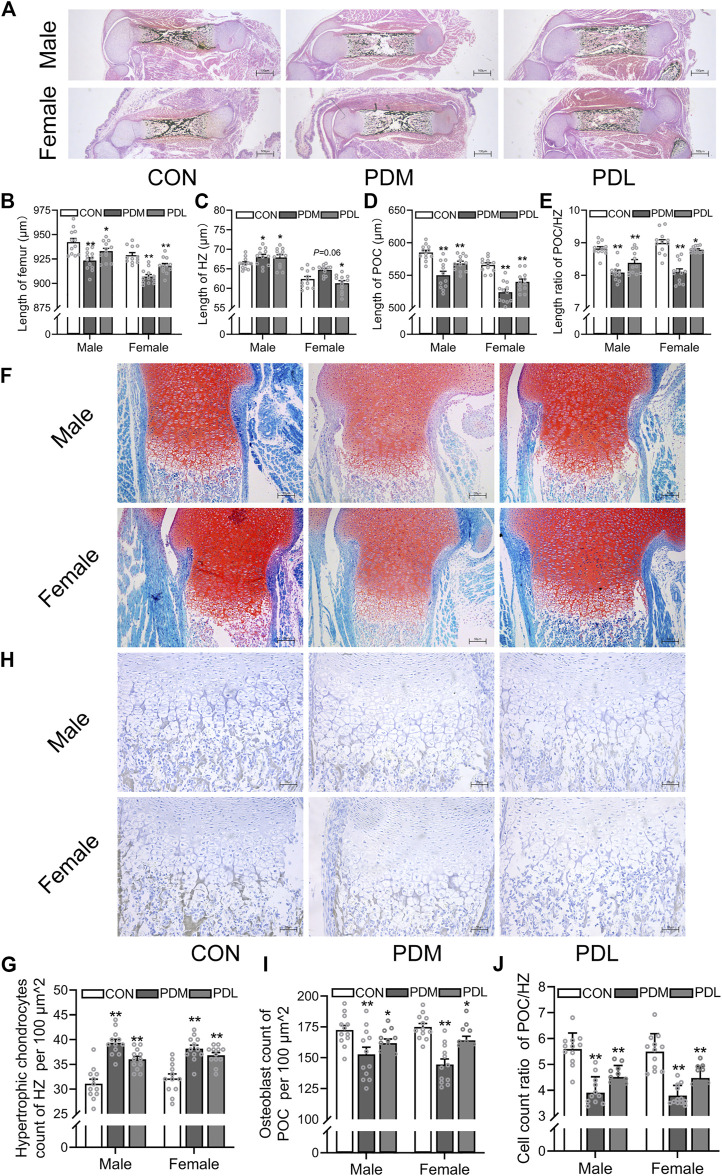
Effects of different stages of dexamethasone exposure on bone development and chondrocyte-to-osteoblast trans-differentiation in fetal mice. **(A)**. Typical images of vonkossa staining in fetal bone tissue; **(B)**. Quantitative analysis of fetal femur length; **(C)**. Quantitative analysis of the width of hypertrophy zone (HZ) in growth plate; **(D)**. Quantitative analysis of the length of the primary ossification center (POC); **(E)**. The ratio between the length of POC and the width of HZ of growth plate cartilage; **(F)**. Typical images of fetal bone tissue stained with saffron O and solid green; **(G)**. Analysis of hypertrophic chondrocytes number in growth plate cartilage; **(H)**. Typical images of alkaline phosphatase (ALP) in fetal bone tissue; **(I)**. Analysis of osteoblasts in the POC of fetal femur; **(J)**. The relative ratio of the osteoblasts number in the POC to the number of hypertrophic cells in the growth plate cartilage. ^*^ *p* < 0.05*,* ^**^ *p* < 0.01*,* CON *v.s.*PDM or PDL. CON, control; PDM, dexamethasone exposure in the middle-late trimester; PDL, dexamethasone exposure in the late trimester.

Next, we investigated the effects of different stages of PDE on bone development and trans-differentiation of growth plate chondrocytes into osteoblasts in fetal mice. Pregnant mice were treated with dexamethasone (0.8 mg/kg) at different gestational stages (GD14-15 and GD16-17) according to the clinical dexamethasone protocol. The pregnant mice were sacrificed on GD18, and the fetal bone tissues were obtained for morphological analysis. We found that dexamethasone exposure at different gestational stages shortened the femur length in both female and male fetal mice, and this reduction was more obvious in the second and third trimesters (GD14-15) (*p <* 0.05, *p <* 0.01, Figures 3A,B). Then, we quantitatively analyzed the length of HZ and POC in fetal mice. The results showed that PDE increased the length of HZ in growth plate cartilage but inhibited the formation of POC, which was more significant in the second and third trimester (GD14-15) (*p <* 0.05, *p <* 0.01, Figures 3C,D). Meanwhile, dexamethasone exposure at different stages of pregnancy reduced the length ratio of POC to HZ (*p <* 0.05, *p <* 0.01, Figure 3E). Furthermore, we found that PDE increased the number of hypertrophic chondrocytes in the growth plate cartilage (*p <* 0.01, Figures 3F,G). We also observed that different gestational stages of dexamethasone exposure reduced the number of osteoblasts in POC (*p <* 0.01, Figures 3H,I). The cell number ratio of hypertrophic chondrocytes to osteoblasts was increased by dexamethasone exposure in both male and female fetal mice, especially in the second and third trimesters (GD14-15) (*p <* 0.01, Figure 3J). In conclusion, the trans-differentiation inhibition of hypertrophic chondrocytes to osteoblasts contributed to the development retardation of the long bone in fetal mice caused by PDE.

### 3.3 Effects of different courses of dexamethasone exposure on bone development and chondrocyte-to-osteoblast trans-differentiation in fetal mice

**FIGURE 4:**
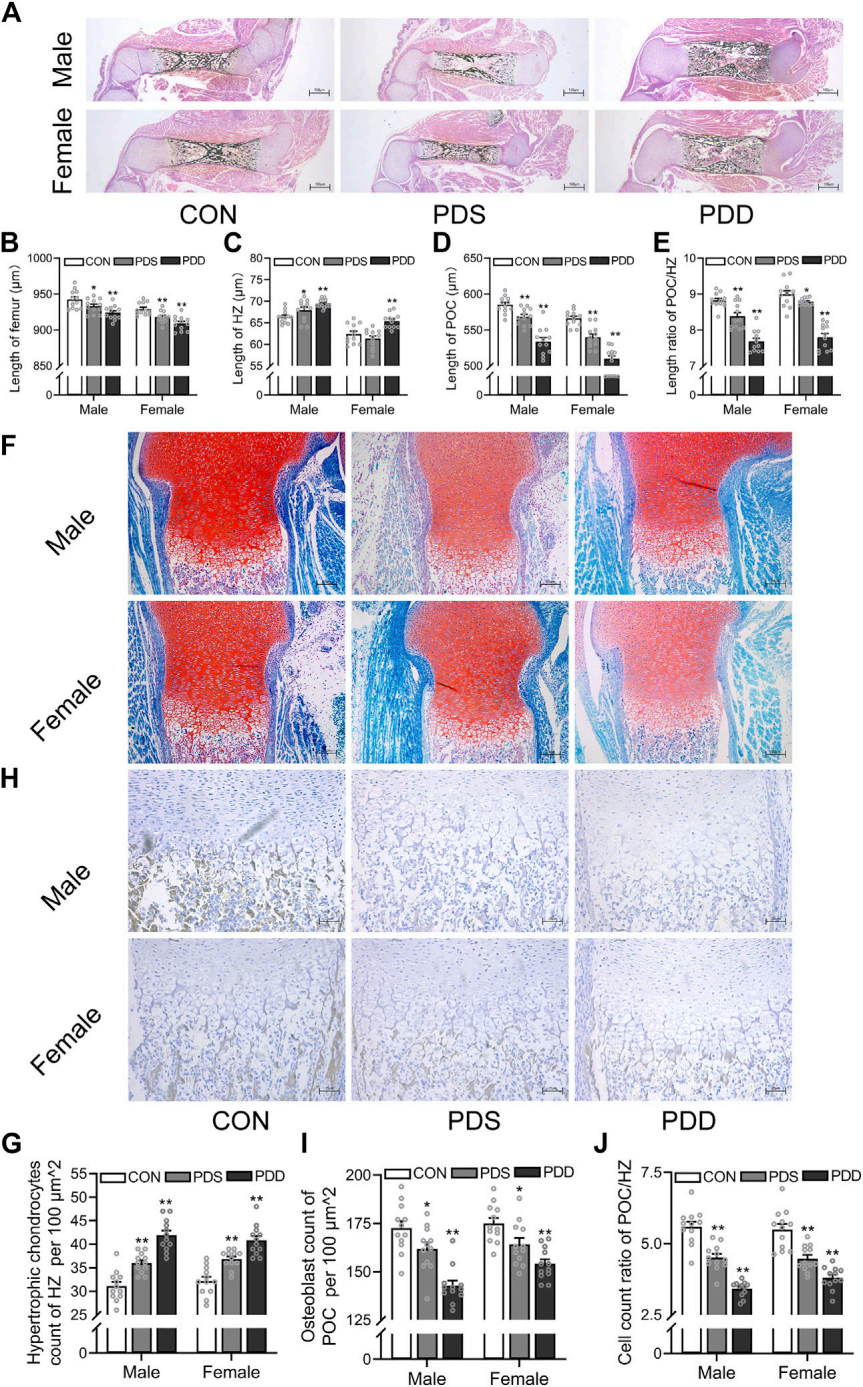
Effects of different courses of dexamethasone exposure on bone development and chondrocyte-to-osteoblast trans-differentiation in fetal mice. **(A)**. Typical images of vonkossa staining in fetal bone tissue; **(B)**. Quantitative analysis of fetal femur length; **(C)**. Quantitative analysis of the width of hypertrophy zone (HZ) in growth plate; **(D)**. Quantitative analysis of the length of primary ossification center (POC); **(E)**. The ratio between the length of POC and the width of HZ in growth plate cartilage; **(F)**. Typical images of fetal bone tissue stained with saffron O and solid green; **(G)**. Analysis of hypertrophic chondrocytes number in growth plate cartilage; **(H)**. Typical images of alkaline phosphatase (ALP) staining in fetal bone tissue; **(I)**. Analysis of osteoblasts in the POC; **(J)**. The relative ratio of the number of osteoblasts in POC to the number of hypertrophic cells in growth plate cartilage. ^*^ *p* < 0.05*,* ^**^ *p* < 0.01*,* CON *v.s.* PDS or PDD. CON, control; PDS, single course of dexamethasone exposure during pregnancy; PDD, double course of dexamethasone exposure during pregnancy.

Then, we investigated the effects of different courses of PDE on bone development and trans-differentiation of growth plate chondrocytes into osteoblasts in fetal mice. The pregnant mice were treated with a single course (GD14-15) or double courses (GD14-15, GD16-17) of dexamethasone (0.8 mg/kg). On GD18, the pregnant rats were sacrificed and the fetal bone tissues were obtained for morphological analysis. We found that different courses of PDE shortened the femur length in both female and male fetal mice, and this reduction was more obvious in double courses (*p <* 0.05, *p <* 0.01, Figures 4A,B). Meanwhile, PDE increased the length of HZ in both female and male fetal mice, and the increase was more significant in double courses of dexamethasone treatment (*p <* 0.05, *p <* 0.01, Figure 4C). Moreover, PDE inhibited the formation of POC, especially in the group of double courses (*p <* 0.01, Figure 4D). The length ratio of the POC to HZ was reduced by different courses of PDE (*p <* 0.05, *p <* 0.01, Figure 4E). Furthermore, we observed that PDE significantly increased the number of hypertrophic chondrocytes in the growth plate cartilage (*p <* 0.01, Figures 4F,G). At the same time, dexamethasone exposure caused a decrease in the number of osteoblasts in POC (*p <* 0.01, Figures 4H,I). However, PDE decreased the cell number ratio of hypertrophic chondrocytes to osteoblasts (*p <* 0.05, *p <* 0.01, Figure 4J). Collectively, the trans-differentiation inhibition of hypertrophic chondrocytes to osteoblasts participated in the bone development retardation caused by PDE, which is more significant in the double courses of dexamethasone exposure.

### 3.4 Effects of different doses, stages, and courses of dexamethasone exposure on the expression of Col10 and Runx2 in growth plate cartilage

**FIGURE 5:**
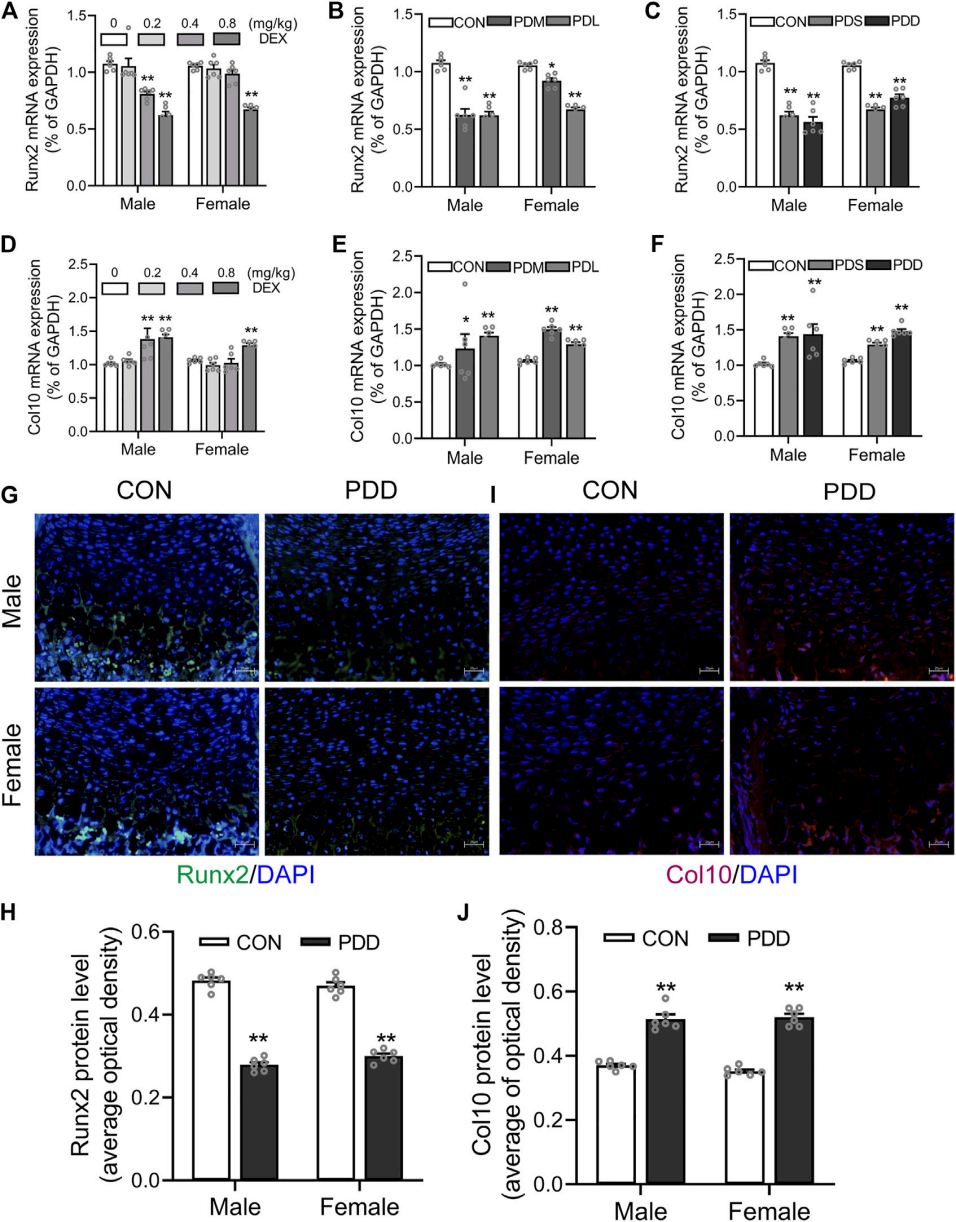
Effects of different doses, stages, and courses of dexamethasone exposure during pregnancy on the Runx2 and Col10 expression in growth plate cartilage. **(A)**. The mRNA expression of Runx2 in different doses of dexamethasone group; **(B)**. The mRNA expression of Runx2 in different stages of dexamethasone group; **(C)**. The mRNA expression of Runx2 in different courses of the dexamethasone group; **(D)**. The mRNA expression of Col10 in different doses of the dexamethasone group; **(E)**. The mRNA expression of Col10 in different stages of dexamethasone group; **(F)**. The mRNA expression of Col10 in different courses of dexamethasone group; **(G)**. Typical immunofluorescence images of Runx2 in fetal bone tissue; **(H)**. Typical immunofluorescence images of Col10 in fetal bone tissue; **(I)**. The protein level of Runx2 in different courses of dexamethasone group; **(J)**. The protein level of Col10 in different courses of the dexamethasone group. ^*^ *p* < 0.05*,* ^**^ *p* < 0.01*,* Control *v.s.* dexamethasone treatment group. CON, control; PDS, single course of dexamethasone exposure during pregnancy; PDD, double course of dexamethasone exposure during pregnancy; PDM, dexamethasone exposure in the middle-late trimester; PDL, dexamethasone exposure in the late trimester; GAPDH, glyceraldehyde 3-phosphate dehydrogenase; Runx2, Runt-related transcription factor 2; Col10, collagen type 10.

We further examined the expression of osteogenic transcription factor Runx2 and the marker gene Col10 of hypertrophic chondrocytes in growth plate cartilage. The results showed that prenatal exposure to dexamethasone downregulated the mRNA expression of Runx2 in the growth plate cartilage in a dose, stage, and course manner (*p <* 0.05, *p <* 0.01, Figures 5A–C). However, the mRNA expression of Col10 was increased by PDE in a dose, stage, and course manner (*p <* 0.05, *p <* 0.01, Figures 5D–F). The protein levels of Runx2 and Col10 also showed the same pattern with the mRNA expression (*p <* 0.01, Figures 5G–J). These above changes in Runx2 and Col10 expression caused by dexamethasone exposure were more significant in the high dose, early stage, and double courses groups. This indicated that the decreased expression of Runx2 and increased expression of Col10 contributed to the trans-differentiation inhibition of hypertrophic chondrocytes to osteoblasts caused by PDE.

### 3.5 Effects of dexamethasone treatment *in vitro* on the trans-differentiation of primary growth plate chondrocytes into osteoblasts

**FIGURE 6:**
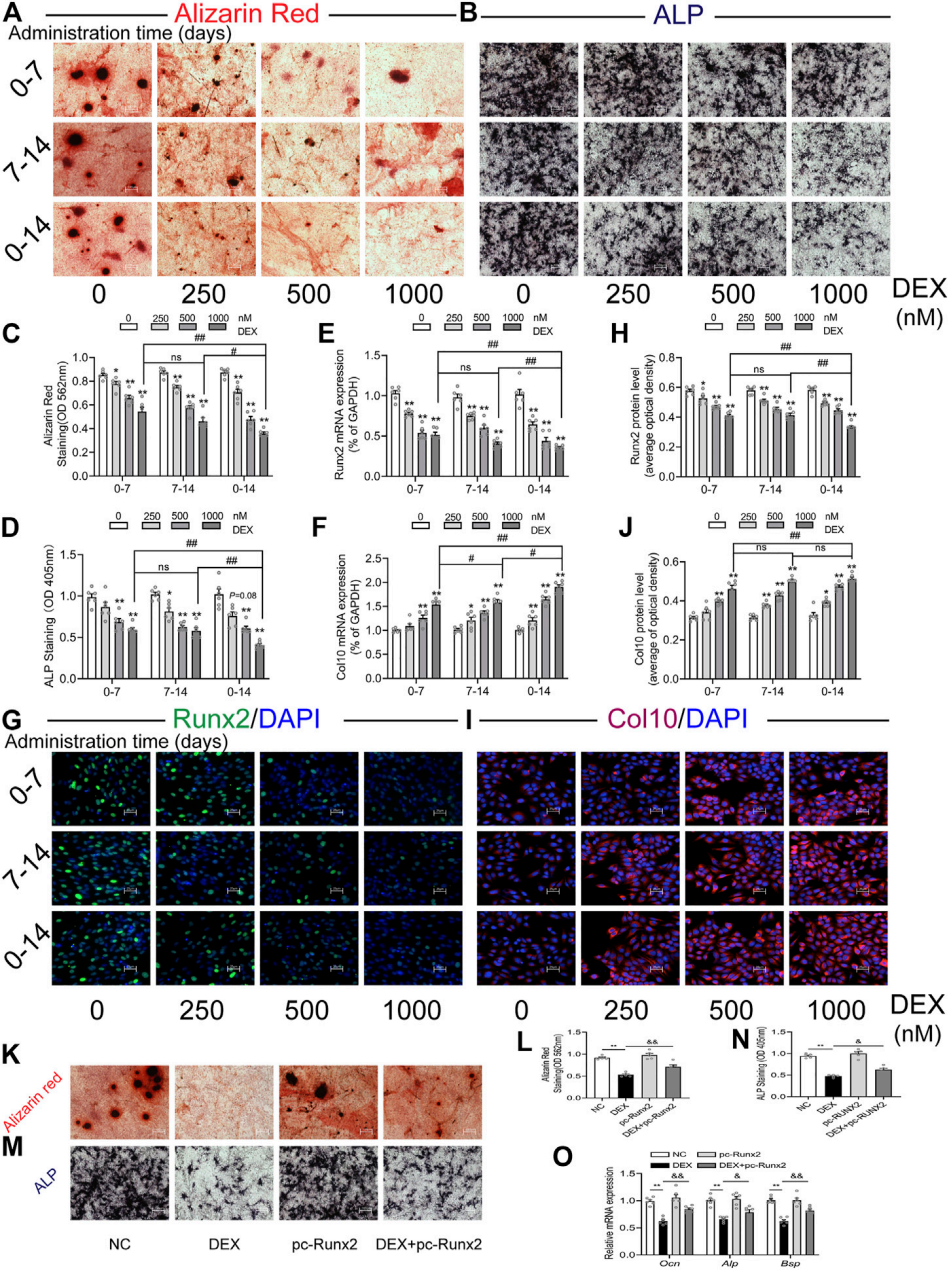
Effects of different times and concentrations of dexamethasone on trans-differentiation of primary growth plate chondrocytes into osteoblasts. **(A)**. Typical images of alizarin red staining; **(B)**. Typical images of alkaline phosphatase (ALP) staining; **(C)**. Quantitative analysis of alizarin red staining; **(D)**. Quantitative analysis of ALP staining; **(E)**. The mRNA expression of Runx2; **(F)**. The mRNA expression of Col10; **(G)**. Typical immunofluorescence images of Runx2; **(H)**. Typical immunofluorescence images of Col10; **(I)**. Quantitative analysis of Runx2 protein level; **(J)**. Quantitative analysis of Col10 protein level; **(K)**. Alizarin red staining after treatment with dexamethasone and Runx2 overexpression plasmid; **(L)**. Quantitative analysis of alizarin red staining after treatment with dexamethasone and Runx2 overexpression plasmid; **(M)**. ALP staining after treatment with dexamethasone and Runx2 overexpression plasmid; **(N)**. Quantitative analysis of ALP staining after treatment with dexamethasone and Runx2 overexpression plasmid; **(O)**. Expression of marker genes for osteogenic differentiation after treatment with dexamethasone and Runx2 overexpression plasmid ^*^ *p* < 0.05*,* ^**^ *p* < 0.01*,* Control vs. dexamethasone treatment group. ^#^ *p* < 0.05*,* ^*##*^ *p* < 0.01*,* compared with different times of dexamethasone treatment. ^&^ *p* < 0.05*,* ^*&* *&*^ *p* < 0.01*,* DEX treatment group *v.s.* DEX + pc-Runx2 treatment group. DEX, dexamethasone; GAPDH, glyceraldehyde 3-phosphate dehydrogenase; Runx2, Runt-related transcription factor 2; Col10, collagen type 10.

Finally, we established a cell trans-differentiation model *in vitro* to determine the effects of dexamethasone on the trans-differentiation of growth plate chondrocytes into osteoblasts. The results showed that the growth plate chondrocytes treated with different concentrations of dexamethasone (250, 500, 1,000 nM) significantly inhibited their osteogenic differentiation in a concentration-dependent manner (*p* < 0.05, *p* < 0.01, Figures 6A–D). Meanwhile, at the same concentration of dexamethasone (1,000 nM), dexamethasone treatment at 0–14 days inhibited the trans-differentiation of growth plate chondrocytes into osteoblasts more obviously than dexamethasone treatment at 0–7 days and 7–14 days (*p* < 0.05, *p* < 0.01, Figures 6A–D). After dexamethasone treatment *in vitro*, the mRNA and protein expression of Runx2 were significantly decreased, while the mRNA and protein expression of Col10 were significantly increased (*p* < 0.05, *p* < 0.01, Figures 6E–J). However, overexpression of Runx2 in growth plate chondrocytes partially reversed dexamethasone-induced inhibition of chondrocyte-to-osteoblast trans-differentiation *in vitro* (*p* < 0.05, *p* < 0.01, Figure 6K–O). These results indicated that different concentrations and times of dexamethasone inhibited the trans-differentiation of primary growth plate chondrocytes into osteoblasts.

## 4 Discussion

The classic clinical use of antenatal dexamethasone to promote lung maturation and reduce the incidence of respiratory distress syndrome in preterm infants is as follows: dexamethasone (6 mg) is injected intramuscularly every 12 h for a total of 4 doses. The optimal time to promote fetal lung maturation with a single course of prenatal dexamethasone is between 24 h and 7 days after treatment (Peaceman et al., 2005). Multiple courses of dexamethasone are used to reduce perinatal mortality in women who are prone to or at high risk for preterm birth and do not deliver within 7 days after a single course of dexamethasone (Roberts et al., 2017). Multiple courses of dexamethasone treatment included repeated administration of dexamethasone weekly and repeated administration until delivery if the delivery did not occur 1 week after administration of dexamethasone (Sweet et al., 2019). To observe the effects of different doses of PDE on long bone development in mice and the role of chondrocyte-to-osteoblast trans-differentiation in this occurrence, we set up three doses of dexamethasone treatment (0.2, 0.4, 0.8 mg/kg) based on pregnant women with the body weight of 60 kg and clinical therapeutic dose. Meanwhile, pregnant mice are exposed to dexamethasone in the second and third-trimester (GD14-15), and the third trimester (GD16-17), or treated with double courses of dexamethasone (GD14-15, GD16-17) to investigate the effects of different stages and courses of dexamethasone exposure. Therefore, the exposure dose, stage, and course of dexamethasone in this study are in line with the clinical treatment status and social reality, which has certain theoretical and clinical practical significance for exploring the phenomenon and potential mechanism of dexamethasone on bone development toxicity of offspring.

Epidemiology studies revealed that prenatal or early postnatal administration of dexamethasone caused adverse effects in offspring, such as low birth weight and dysplasia of long bones, which are closely related to the decline of bone quality and mechanical properties in the early postnatal period (Shi et al., 2018; Cao et al., 2021). In the present study, we confirmed that PDE caused development retardation of the long bone in fetal mice based on animal experiments, especially in the high-dose, early stage, and double courses dexamethasone exposure group and this development retardation in males was more significant than in females. Recent studies have shown that during endochondral ossification, most growth plate chondrocytes become mature and do not enter apoptosis after hypertrophy, but directly transdifferentiate into osteoblasts (Pazzaglia et al., 2020; Matsushita et al., 2020; Ballock and O’Keefe, 2003). Moreover, the trans-differentiation of hypertrophic chondrocytes into osteoblasts in the growth plate plays an important role in the process of bone development [18]. However, whether dexamethasone can affect the trans-differentiation of growth plate chondrocytes into osteoblasts and the role of this effect in dexamethasone-induced fetal bone dysplasia is still unclear. In this study, we found that PDE inhibited the POC formation and increase the length of the HZ in growth plate cartilage. Moreover, PDE increased the number of hypertrophic chondrocytes in the growth plate cartilage but reduced the number of osteoblasts in the POC. The cell number ratio of osteoblasts to hypertrophic chondrocytes was also reduced by PDE. Conclusively, the trans-differentiation inhibition of hypertrophic chondrocytes to osteoblasts participated in the bone development retardation caused by PDE, which is more significant in the high-dose, early stage, and double courses of dexamethasone exposure.

Runx2, a member of the Runx family of transription factors, is expressed in both growth plate chondrocytes and osteoblasts (Hinoi et al., 2006). Recent studies have shown that Runx2 can promote the terminal differentiation of growth plate chondrocytes and is an important transcription factor regulating the trans-differentiation of hypertrophic chondrocytes into osteoblasts (Bond et al., 2011; Lee et al., 2012). Knocking out Runx2 in hypertrophic chondrocytes of the growth plate could lead to the disorder of trans-differentiation of hypertrophic chondrocytes into osteoblasts, and cause retardation of bone development in neonatal mice and low bone mass in adult mice (Shea et al., 2017). Therefore, maintaining the stability of Runx2 expression is essential for hypertrophic chondrocytes into osteoblasts trans-differentiation. Therefore, we further detected the expression of osteogenic transcription factor Runx2 and the marker gene Col10 for hypertrophic chondrocytes in growth plate cartilage. The results showed that different doses, stages, and courses of dexamethasone treatment during pregnancy inhibited the expression of Runx2 in the growth plate cartilage of fetal rats, but increase the expression of Col10. *In vitro*, we established a trans-differentiation model of growth plate chondrocytes into osteoblasts to determine the effect of dexamethasone on the trans-differentiation of growth plate chondrocytes into osteoblasts. The results showed that treatment with different concentrations of dexamethasone significantly inhibited the osteogenic trans-differentiation of growth plate chondrocytes in a concentration and time-dependent manner. Meanwhile, after dexamethasone treatment *in vitro*, the expression of Runx2 was significantly decreased, while the expression of Col10 was significantly increased. Therefore, we confirmed that dexamethasone inhibited the trans-differentiation of growth plate chondrocytes into osteoblasts *in vivo* and *in vitro*.

## 5 Conclusion

In summary, this study confirmed that PDE at different doses, stages, and courses caused trans-differentiation disorder of growth plate chondrocytes to osteoblasts in fetal mice, which contributed to the dysplasia of long bones in fetal mice, especially in high doses, early stage, and double courses of dexamethasone exposure. Meanwhile, this above manifestation of male fetal mice is more typical than that of female fetal mice. This study also confirmed *in vivo* and *in vitro* that dexamethasone inhibited trans-differentiation of growth plate chondrocytes into osteoblasts by downregulating Runx2 expression and increasing Col10 expression. Therefore, this study revealed the phenomenon and mechanism of fetal bone dysplasia caused by PDE from the new perspective of trans-differentiation disorder of growth plate chondrocytes to osteoblasts, which provided the experimental and theoretical basis for the rational use of dexamethasone in clinical practice and the exploration of early therapeutic strategy.

## Data Availability

The raw data supporting the conclusions of this article will be made available by the authors, without undue reservation.
